# A Case of Reflex Otalgia

**Published:** 1887-01

**Authors:** S. Latimer Phillips

**Affiliations:** Savannah, Ga., Formerly House Surgeon to the Presbyterian Eye, Ear and Throat Charity Hospital, Baltimore, Md.


### Clinic Reports.

### A CASE OF REFLEX OTALGIA.

BY S. LATIMER PHILLIPS, M. D., SAVANNAH, GA., FORMERLY HOUSE SURGEON TO THE PRESBYTERIAN EYE, EAR AND THROAT CHARITY HOSPITAL, BALTIMORE, MD.

Miss A. H., æt. 35, of nervous temperament and rather plethoric, consulted me July 12th, 1884, while I was in Virginia on my summer vacation, for an "ear ache." She complained of deep-seated pain in right side of head, which she referred to the tympanic cavity, and extending along the region of the third branch of the fifth nerve. The pain would come on in paroxysms at intervals of an hour or so and then again she would not have it for days, it always being more exaggerated when she was exposed to cold or dampness, but on removal from these influences it would become less, leaving a soreness in the parts.

On examining her hearing with the watch, I found it normal; membrana tympani in perfect condition; no tinnitus aurium. Thinking it might be a reflex otalgia, I examined her teeth for caries, but found all on right side in perfect state of preservation, except one bicuspid, which had in it an amalgam filling, and which appeared to be keeping the tooth. Knowing that sometimes fillings are put in upon diseased nerves, and as this might be the case here, I advised her to go to a competent dentist and have the tooth examined. She did so. He told her that all the teeth were all right. But having no history of malaria or rheumatism, and her high social position excluding all ideas of syphilis, I still suspected the tooth to be at fault and told her to keep it under observation. About that time the patient went away to visit some friends, and still suffering, put herself under the treatment of their family physician, who prescribed for her quinine and iron, which she thought improved her condition for a while.

She returned home on the 20th of August and I again saw her. She was still suffering, even more than before. The pain in the ear now was not so great, the most severe being in the ophthalmic division of the fifth and the inferior maxillary. There was considerable swelling about the right lower jaw, the pain coming on in severe paroxysms. August 27th, patient suffered much previous night, but was relieved by having an incision made in the jaw at the most painful point, which was just at the apex of the root of the bicuspid with the filling. Shortly a little pus oozed from the opening, all pain disappeared and soon swelling had subsided. I saw patient for the last time on the 29th of August. She was feeling quite well, having got relief from all suffering. I then returned to Baltimore. In March, 1885, patient stopped in to see me. Was in perfect condition and had never had a return of the otalgia.

---

FERNANDEZ ON A NEW ANTI-RABIES INOCULATION—Dr. Fernandez, of Barcelona, claims to have discovered a new vaccine to preserve men and animals from the contagion of rabies. He has collected a great number of observations, showing that dogs bitten accidentally by vipers are never affected by rabies, either spontaneously or when bitten by affected animals. He has made certain direct experiments, inoculating dogs with a small quantity of the viper's poison. After inoculation the animals were ill for four or five days with symptoms of slight fever, prostration and more or less profound somnolence. He maintains that the animals thus operated on are protected from rabies, and that neither when inoculated with the saliva, nor when bitten by affected animals, do the contract they disease.—*Medical News.*

